# Treatment incidence of and medical utilization for hospitalized subjects with pathologic fractures in Taiwan-Survey of the 2008 National Health Insurance data

**DOI:** 10.1186/1472-6963-11-230

**Published:** 2011-09-22

**Authors:** Yi-Hui Lee, Yuan-Nian Hsu, I-Liang Yu, Dinh-Van Phan, Pesus Chou, Chien-Lung Chan, Nan-Ping Yang

**Affiliations:** 1 Institute of Nursing, College of Medicine, National Taiwan University, Taipei, Taiwan; 2 Department of Nursing, School of Nursing, Chang-Gang University, Taoyuan, Taiwan; 3 Department of Medical Research & Department of Orthopedic Surgery, Taoyuan General Hospital, Department of Health, Taoyuan, Taiwan; 4 College of Management, Central Taiwan University of Science and Technology, Taichung, Taiwan; 5 Department of Information Management, Yuan-Ze University, Taoyuan, Taiwan; 6 Community Health Research Center & Institute of Public Health, National Yang-Ming University, Taipei, Taiwan

**Keywords:** Incidence, utilization, pathologic fracture

## Abstract

**Background:**

Almost all studies of pathologic fractures have been conducted based on patients with tumours and hospital-based data; however, in the present study, a nationwide epidemiological survey of pathologic fractures in Taiwan was performed and the medical utilization was calculated.

**Methods:**

All claimants of Taiwan's National Health Insurance (NHI) Program in 2008 were included in the target population of this descriptive cross-sectional study. The registration and inpatient expenditure claims data by admission of all hospitalized subjects of the target population were examined and the concomitant International Classification of Diseases, Ninth Revision, Clinical Modification (ICD-9-CM) diagnosis codes were evaluated and classified into seven major categories of fracture.

**Results:**

A total of 5,244 incident cases of pathologic fracture were identified from the 2008 hospitalized patient claims data. The incidence of pathologic fracture of the humerus, distal radius/ulna, vertebrae, femoral neck, other part of the femur, and tibia/fibula was 0.67, 0.08, 10.58, 1.11, 0.56, and 0.11 per 100,000 people, respectively, and patients with those fractures were hospitalized for 43.9 ± 42.9, 31.1 ± 32.9, 29. 4 ± 34.4, 43.3 ± 41.2, 42.4 ± 38.1, and 42.0 ± 32.8 days, respectively, incurring an average medical cost of US$11,049 ± 12,730, US$9,181 ± 12,115, US$6,250 ± 8,021, US$9,619 ± 8,906, US$10,646 ± 11,024, and US$9,403 ± 9,882, respectively. The percentage of patients undergoing bone surgery for pathologic fracture of the humerus, radius/ulna, vertebrae, femoral neck, other part of the femur, and tibia/fibula was 31.2%, 44.4%, 11.3%, 46.5%, 48.4%, and 52.5% respectively.

**Conclusions:**

Comparing Taiwan to other countries, this study observed for Taiwan higher medical utilization and less-aggressive surgical intervention for patients hospitalized with pathologic fractures.

## Background

The skeleton is the third most common target of metastatic cancer and can be one of the earliest sites affected. Ultimately, 60-84% of all cases of metastatic disease invade the bone, and approximately 70% of these patients experience bone pain [1]. Patients with metastatic cancer involving the bone are also at increased risk of fractures, spinal cord compression, hypercalcemia and immobility, resulting in substantial medical-associated morbidities [2]. Patients with long-bone or pelvic-girdle metastases are indicated for surgical treatment if they suffer a pathologic fracture, impending fracture or intractable pain [3], and surgical treatment of spinal metastases is indicated for patients with spinal instability or spinal cord compression [4]. Therefore, the issue of pathologic fractures should be considered more important than normal traumatic fractures in the orthopaedic field of the treatment of fragility fractures.

Most pathologic fracture studies have been conducted based on tumour-associated patients and hospital-based data, and no nationwide epidemiological survey of pathologic fractures has been performed to date. This study therefore aimed to perform a nationwide epidemiological survey of pathologic fractures in Taiwan independent of cause and calculate the medical utilization, which could be of use to policymakers in Taiwan in redistributing medical resources and promoting more aggressive treatment.

## Methods

### Source, security, and quality control of data

Taiwan launched a single-payer NHI Program in 1995, and as of 2007, 22.60 million Taiwanese people of a total population of 22.96 million were enrolled in this program; foreigners in Taiwan are also eligible for inclusion. In order to respond to current and emerging health issues rapidly and effectively, in cooperation with the National Health Insurance Bureau (NHIB), the National Health Research Institute (NHRI) established a nationwide research database, and the NHIB established a uniform system to control the quality of medical services and coding.

The NHRI safeguards the privacy and confidentiality of patients included in the database and routinely transfers health insurance data from the NHIB to enable health researchers to analyse and improve the health of Taiwan's citizens. The National Health Insurance (NHI) database contains registration files and original claims data for reimbursement, and access to the National Health Insurance Research Database (NHIRD), which was derived from this system by the NHIB and is maintained by the NHRI, is provided to scientists in Taiwan for research purposes.

### Data protection and permission

Any data in the NHIRD that could be used to identify patients or care providers, including medical institutions and physicians, are scrambled before being sent to the NHRI for database inclusion, and the data are further scrambled before being released to researchers. Theoretically, it is impossible to query the data alone to identify individuals at any level using this database. The protocol of this study was evaluated by the NHRI, who gave their agreement to the planned analysis of the NHIRD (Application and Agreement Number: 99005). This study was also approved by the Institutional Review Board (IRB) of Taoyuan General Hospital, which has been certificated by the Department of Health, Taiwan (IRB Approval Number: TYGH098033).

### Data selection and definition of pathologic fractures

All claimants of Taiwan's NHI Program at any time during 2008 were included in the target population of this descriptive cross-sectional study. In order to investigate the treatment incidence of pathologic fractures among hospitalized subjects of the target population, the registration and claims data of inpatient expenditure by admission were examined and the concomitant ICD-9-CM diagnosis codes were evaluated and classified into seven major categories. Pathologic fracture of the humerus was defined as the diagnosis code 733.11 and pathologic fracture of the distal radius/ulna was coded 733.12. The third category, pathologic fracture of vertebrae, was coded 733.13. Pathologic fractures of the neck of the femur, any other part of the femur, and the tibia/fibula were coded 733.14, 733.15 and 733.16, respectively. The seventh category, pathologic fractures of unspecified or other sites, included all other cases and was coded 733.1, 733.10 or 733.19.

### Statistics

The method of back-projection was used to estimate the unobserved past incidences of various pathologic fractures, which are expressed as the proportions of the target population in Taiwan. The independent t-test and Pearson's chi-square test were used for simple descriptive comparison of differences in the indicators of medical utilization and bony surgery rates between genders.

## Results

### Treatment incidence of pathologic fractures of various locations and associated metabolic disorders

According to the previously-defined selection criteria, 3,094 subjects who were hospitalized for pathologic fracture in 2008 were identified. Of the 3,094 studied subjects, 5,244 incident cases of various categories of pathologic fracture were identified within one year (i.e., in 2008, Taiwan) (Table 1), which included 3,003 recognized cases of fracture in six major locations and 2,241 almost concomitant with the above but unspecified cases. By dividing the number of cases by the number of beneficiaries of the same age and gender in 2008, the estimated incidence of pathological fracture requiring hospitalization in Taiwan categorized by fracture location was calculated. The treatment incidence of most of the variously-located pathologic fractures increased with age for both genders, and this trend was very obvious for fractures of the vertebrae and femoral neck. In general, the estimated incidence of pathologic fracture in Taiwan was 22.89 per 100,000 people based on the nationwide health insurance hospitalization data, and the individual incidence of fracture of the humerus, distal radius/ulna, vertebrae, femoral neck, other femoral location, and tibia/fibula was 0.67, 0.08, 10.58, 1.11, 0.56, and 0.11 per 100,000 people, respectively. Worthy of note, a high proportion of unspecified or alternatively-located pathologic fractures was noted, the estimated incidence being 9.78 per 100,000 people.

**Table 1:** Treatment incidence of pathologic fracture cases categorized by anatomic location in Taiwan, 2008 (valid *n *= 5244)

	Total		Inc.* for those		Inc.* for those		Inc.* for those	
	**enrolled cases**		**aged 0-19.9 **		**aged 20-49.9 **		**aged 50 or over **	
Category of fracture	Cases	Inc. *	Female	Male	Female	Male	Female	Male
Pathologic fracture of humerus (733.11)	154	0.67	0.04	0.21	0.30	0.18	1.82	2.03
Pathologic fracture of distal radius and ulna (733.12)	18	0.08	0.00	0.04	0.05	0.05	0.25	0.10
Pathologic fracture of vertebrae (733.13)	2424	10.58	0.04	0.14	1.91	1.33	41.97	29.29
Pathologic fracture of neck of femur (733.14)	254	1.11	0.04	0.14	0.39	0.31	3.63	3.08
Pathologic fracture of other specified part of femur (733.15)	128	0.56	0.12	0.14	0.19	0.22	1.72	1.41
Pathologic fracture of tibia and fibula (733.16)	25	0.11	0.00	0.04	0.04	0.09	0.28	0.26
Pathologic fracture, other specified or unspecified sites (733.1,733.10, 733.19)	2241	9.78	0.27	0.53	2.49	2.21	33.36	29.06

*Inc.: treatment incidence, 1/100,000.

### Medical utilization for the various identifiably-located pathologic fractures and concomitant bony surgery

Table 2 shows the average medical utilization for patients hospitalized with identifiably-located pathologic fractures in Taiwan. For humeral pathologic fractures, the average hospital stay was 43. 9 ± 42.9 days and the associated direct medical cost was US$11,049 ± 12,730 (currency conversion rate: US$1 = NT$32). For distal radial/ulnar, vertebral, femoral neck, other part of the femur, and tibial/fibular pathologic fractures, patients stayed in hospital for 31.1 ± 32.9, 29.4 ± 34.4, 43.2 ± 41.2, 42.4 ± 38.1 and 42.0 ± 32.8 days, respectively, and the associated direct medical cost was US$9,181 ± 12,114, US$6,250 ± 8,021, US$9,619 ± 8,906, US$10,646 ± 11,024 and US$9,403 ± 9,882, respectively. The differences in medical utilization between genders were also analysed, and a significant finding was that for cases of pathologic fracture of the distal radius/ulna or vertebrae, more medical resources (in terms of a longer length of stay and a greater medical cost) were required for male patients.

**Table 2:** Medical utilization for subjects with a pathologic fracture of an identifiable location in Taiwan, 2008 (valid n = 3003)

	Medical Cost ($NT), mean, (S.D.)										Length of Stay (days), mean, (S.D.)									
				
	In general			Female			Male				In general			Female			Male			
				
Fracture location	cases	mean	(S.D.)	*cases*	mean	(S.D.)	*cases*	mean	(S.D.)	P *	*cases*	mean	(S.D.)	*cases*	mean	(S.D.)	*cases*	mean	(S.D.)	P *
**Pathological Fx of Humerus**	***154***	353,575	407,372	***76***	360,005	346,344	***78***	347,309	461,335	0.357	***154***	43.9	42.9	***76***	48.3	45.7	***78***	39.6	39.8	0.402
**Pathological Fx of Distal Radius/Ulna**	***18***	293,803	387,673	***11***	178,740	210,325	***7***	474,616	538,219	0.009	***18***	31.1	32.9	***11***	21.0	19.0	***7***	47.0	44.5	0.020
**Pathological Fx of Vertebrae**	***2424***	200,015	256,668	***1,451***	186,175	245,394	***973***	220,654	271,435	0.005	***2424***	29.4	34.4	***1,451***	26.3	30.3	***973***	33.9	39.4	0.000
**Pathological Fx of Neck of Femur**	***254***	307,807	284,991	***139***	303,879	248,781	***115***	312,555	324,480	0.324	***254***	43.3	41.2	***139***	45.7	42.7	***115***	40.4	39.5	0.327
**Pathological Fx of Other Part of Femur**	***128***	340,675	352,755	***69***	350,874	394,534	***59***	328,747	299,516	0.270	***128***	42.4	38.1	***69***	43.5	42.4	***59***	41.3	32.7	0.165
**Pathological Fx of Tibia and Fibula**	***25***	300,888	316,227	***11***	290,137	309,024	***14***	309,335	333,140	0.451	***25***	42.0	32.8	***11***	43.6	26.5	***14***	40.6	38.0	0.119

S.D.: standard deviation

*Descriptive comparison of differences between genders was performed by the independent *t*-test.

Finally, the requirement for surgical intervention in hospitalized pathologic fracture cases was investigated (Table 3), and the percentage of cases in which bony surgery was performed for pathologic fractures of the humerus, radius/ulna, vertebrae, femoral neck, other part of the femur, and tibia/fibula was 31.2%, 44.4%, 11.3%, 46.5%, 48.4%, and 52.5%, respectively. The difference in the bony surgery rate for each fracture category between genders was investigated, and no statistically significant differences were noted. The bony surgery procedures were stratified into instrument fixation, prosthesis application, bone biopsy or excision, and bone-grafting or other bone plastic procedures, and surgical interventions in the spinal area were further classified as anterior or posterior-lateral fusion procedures.

**Table 3:** Distribution of surgical intervention for pathologic fractures of an identifiable location in Taiwan, 2008 (valid n = 3003)

	Pathologic Fx of Humerus (*n *= 154)	Pathologic Fx of Distal Radius/Ulna (*n *= 18)	Pathologic Fx of Vertebrae (*n *= 2424)	PathologicFx of Neck of Femur (*n *= 254)	Pathologic Fx of Other Part of Femur (*n *= 128)	Pathologic Fx of Tibia and Fibula (*n *= 25)
	
	*n *(%)	*n *(%)	*n *(%)	*n *(%)	*n *(%)	*n *(%)
Bony surgery performed?						
Yes	48 (31.2%)	8 (44.4%)	273 (11.3%)	118 (46.5%)	62 (48.4%)	13 (52.0%)
No	106 (69.8%)	10 (55.6%)	2151 (88.7%)	136 (53.5%)	66 (51.6%)	12 (48.0%)
Bony surgery strategy:						
1. Instrument fixation	41	8		58	52	13
2. Spine fusion			91* 164**			
3. Prosthesis	3	0		51	5	0
4. Excision, biopsy	39	6	98	46	38	9
5. Bone-grafting, bone plastic surgery	6	5	28	9	11	4

*anterior fusion procedures

**posterior-lateral fusion procedures

Note-There were no statistically significant differences between genders, for bony surgery rates, tested separately for each fracture category.

## Discussion

The exact incidence of bone metastasis is unknown, but of the estimated 1.2 million new cases of cancer diagnosed annually in the US, more than 50% will eventually demonstrate skeletal metastases [5], and it is estimated that 350,000 people die of bone metastases annually in the US [6]. National prevalence estimates were produced from the 2000-2004 MarketScan databases of the USA, and of 396,200 oncology patients, 18,042 (4.6%) were identified as having bone metastasis. Applying the projection methodology, 5.3% of these patients were projected to have bone metastases [7]. This study revealed that the estimated incidence of treated pathologic fracture is 22.89 per 100,000 people annually in Taiwan. More epidemiological cohort surveys of the oncology registry data in Taiwan are needed in order to compare the cumulative incidence of pathologic fracture with that in other countries.

The incidences of pathologic fractures of some specific locations have rarely been reported. One epidemiological survey of 401 fractures of the shaft of the humerus revealed that 8% of the fractures were pathologic [8]. A retrospective 36-year chart review of all patients with metastatic bone disease admitted to a single tertiary orthopaedic referral centre was conducted, which identified 601 females and 580 males (mean: 60 years) with metastatic bone disease, of the femur (28%) and spine (29%) in the main [9]. In the present study of pathologic fractures in Taiwan requiring hospitalization, the spine was found to be the most common location, followed by parts of the femur and the humeral area. However, a high proportion (42.7%) of cases of pathologic fracture at unspecified sites was noted, and, possibly through some administrative encouragement means provided by the Taiwan's health insurance authority, the accuracy of coding should be improved to ensure the validity of the data of Taiwan's health insurance system.

Few studies of the medical burden of bone metastatic disease or pathologic fractures have been performed, but the cost of bone metastasis in cancer patients was estimated to be $12.6 billion dollars per year (17% of the total direct medical cost) in the US from 2000 to 2004 [7]. In the Netherlands, a retrospective cost analysis study associated with bone metastases in patients with prostate cancer showed an average cost of 6,973 Euros per patient to treat skeletal-related events [10]. We calculated in this study the mean direct medical cost and administrative utilization for individual pathologic fracture cases in Taiwan, to compare with individual non-pathologic fracture cases in Taiwan according to Taiwan Diagnoses Related Groups (TW-DRGs), the new payment regulations in Taiwan that have been in place since January 1st 2010. For example, a lower-extremity (excepting the femur) or humeral fracture can be paid US$583-3,109, with an average of 6-8 days in hospital being allowed, and for a hip or femoral fracture, the cost is US$952-3,691, with an average stay of 7-10 days [11]. These values are much lower than those estimated in the present study. However, owing to the scarcity of published literature on this topic, we were not able to adequately compare Taiwanese expenditures against expenditures of other nations, for bone metastasis or for the costs of follow-up treatment for metastatic bony events. Future studies concerning medical economic issues should be encouraged in order to assist public health policymakers in making policy decisions worldwide.

Several decades ago, more conservative treatments for pathologic fractures were accepted. A study evaluating the results of treating seventy-two pathologic long-bone fractures revealed that only 25% of upper-extremity and 57.5% of lower-extremity pathologic fractures were treated with internal fixation, and better outcomes were noted for those treated by internal fixation than those treated by other means [12]. Recently, surgical treatment has been employed for impending or existing pathologic fractures. In a recent prospective study based upon the skeletal metastasis register of the Scandinavian Sarcoma Group, pathologic fracture was found to be an independent negative prognostic factor of one-year survival [13]. Another recent study showed that of 2,776 patients with intracapsular fractures of the proximal femur, 18% of patients were treated non-surgically. Included in the group of patients who were treated conservatively were children, patients with cardiac problems, mental problems, stroke, renal failure, and multiple disseminated malignancies, and patients who chose non-surgical treatment [14]. In comparison with the proportions of cases in which surgical intervention was performed reported in the above-mentioned studies, the surgical rate for pathologic fracture in Taiwan was found in this study to be on a par with that reported for other countries for the upper limbs but lower for the lower limbs. This study took a nationwide overview of pathologic fractures registered in the health insurance inpatient database rather than examining the outcomes of patients referred to hospital for treatment in Taiwan. More conservative treatment in some community hospitals and less adequate referral processes to tertiary treatment centres are possible causes of this difference, and further studies should be performed in the future to investigate this issue.

There were some principal limitations in the present study. This was a one-year cross sectional study investigating the characteristics of pathological fractures requiring hospitalization based on the NHI dataset. Claims data are not linked to the death register databank in Taiwan; therefore, prospective survival analysis for subsequent events, including complications of surgery or death, was not able to be performed. Further information and investigations are needed in the future.

## Conclusion

We observed in our study that medical utilization for pathologic fracture cases was higher in Taiwan compared to that reported for other nations. However, we also noted less-aggressive surgical intervention for patients hospitalized with pathologic fractures in Taiwan, in comparison with other countries.

## Competing interests

This study was funded by Taoyuan General Hospital, Department of Health, Executive Yuan, Taiwan only. All authors declare that they have no conflicts of interest, including directorships, stock holdings or contracts.

## Authors' contributions

The study was designed by YHL, CLC and NPY; data were gathered and analysed by IY, DVP and NPY; the initial draft of the manuscript was written by YHL, YNH and NPY; and the accuracy of the data and analyses was assured by PC, CLC and NPY. All authors participated in the preparation of the manuscript and approved the final version.

## Pre-publication history

The pre-publication history for this paper can be accessed here:

http://www.biomedcentral.com/1472-6963/11/230/prepub
